# Supplementary material for: Blood-Based Biomarker Analysis for Predicting Efficacy of Chemoradiotherapy and Durvalumab in Patients with Unresectable Stage III Non-Small Cell Lung Cancer
Source: Cancers (Basel). 2023 Feb 10;15(4):1151. doi: 10.3390/cancers15041151 (PMC9953836; doi:10.3390/cancers15041151)
Supplement: Supplementary file 1 [file cancers-15-01151-s001.zip › cancers-2193036-supplementary.pdf]

Blood-Based Biomarker Analysis for Predicting Efficacy of Chemoradiotherapy and Durvalumab in Patients with Unresectable Stage III Non-Small Cell Lung Cancer

Cheol-Kyu Park [1], Sung-Woo Lee [2,3], Hyun-Ju Cho [1], Hyung-Joo Oh [1], Young-Chul Kim [1], Yong-Hyub Kim [4], Sung-Ja Ahn [4], Jae-Ho Cho [2,3,5] and In-Jae Oh [1,*]

Supplementary Table S1. Longitudinal monitoring of CTC and PBC counts in patients on concurrent chemoradiotherapy (CCRT) alone and durvalumab consolidation (DC).

	C0	C1	*p*-value (C0 vs C1)	C2	*p*-value (C1 vs C2)	C3	*p*-value (C1 vs C3)	C5	*p*-value (C1 vs C5)
**CTC,** /7.5mL **[Total]**	4.5 (6.5)	14.4 (29.6)	0.335	-	-	-	-	4.9 (9.3)	0.030
CCRT alone (*n* = 27)	2.7 (4.1)	22.7 (41.9)	0.177	-	-	-	-	4.8 (8.7)	0.173
DC (*n* = 20)	6.8 (8.2)	7.7 (9.9)	0.983	5.7 (11.6)	0.277	6.0 (14.6)	0.570	5.0 (10.0)	0.093
**CTC cluster**, /7.5mL **[Total]**	0.8 (1.4)	3.4 (10.4)	0.946	-	-	-	-	0.5 (1.0)	0.379
CCRT alone (*n* = 27)	0.7 (1.5)	6.7 (15.0)	0.507	-	-	-	-	0.5 (1.2)	0.407
DC (*n* = 20)	0.9 (1.3)	0.7 (0.8)	0.550	0.5 (1.1)	0.527	1.0 (3.0)	0.688	0.4 (0.9)	0.562
**ANC,** x10³/μL **[Total]**	6.4 (2.9)	3.4 (1.7)	<0.001	-	-	-	-	4.4 (2.0)	<0.001
CCRT alone (*n* = 27)	6.9 (3.4)	3.6 (2.0)	<0.001	-	-	-	-	4.9 (2.5)	0.001
DC (*n* = 23)	5.9 (2.2)	3.1 (1.2)	<0.001	4.0 (1.7)	0.005	4.0 (1.5)	0.008	3.8 (1.2)	0.005
**ALC,** x10³/μL **[Total]**	1.7 (0.6)	1.0 (0.4)	<0.001	-	-	-	-	1.1 (0.3)	0.393
CCRT alone (*n* = 27)	1.7 (0.6)	1.0 (0.4)	<0.001	-	-	-	-	1.1 (0.3)	0.779
DC (*n* = 23)	1.8 (0.6)	1.0 (0.4)	<0.001	1.1 (0.4)	0.140	1.1 (0.4)	0.313	1.1 (0.4)	0.151
**PLT,** x10³/μL **[Total]**	290.2 (88.6)	209.6 (57.1)	<0.001	-	-	-	-	226.3 (82.8)	0.068
CCRT alone (*n* = 27)	306.7 (92.9)	217.4 (58.3)	<0.001	-	-	-	-	241.5 (87.5)	0.077
DC (*n* = 23)	270.9 (81.0)	200.8 (55.6)	<0.001	222.2 (115.9)	0.422	219.3 (99.2)	0.117	210.4 (76.5)	0.390
**NLR [Total]**	4.44 (4.00)	4.41 (6.76)	0.459	-	-	-	-	4.45 (2.93)	0.003
CCRT alone (*n* = 27)	5.13 (5.11)	5.43 (9.14)	0.694	-	-	-	-	5.05 (3.65)	0.010
DC (*n* = 23)	3.62 (1.85)	3.26 (1.51)	0.484	4.19 (2.84)	0.259	4.04 (2.15)	0.351	3.82 (1.78)	0.126
**PLR [Total]**	188.11 (97.84)	255.61 (223.57)	0.003	-	-	-	-	222.58 (91.36)	0.946
CCRT alone (*n* = 27)	210.12 (115.74)	291.29 (294.81)	0.069	-	-	-	-	236.73 (94.35)	0.351
DC (*n* = 23)	162.28 (64.81)	215.28 (84.19)	0.021	237.93 (186.45)	0.339	224.26 (127.59)	0.823	207.73 (88.03)	0.332

Values are presented as mean (SD). SD, standard deviation; CTC, circulating tumor cell; PBC, peripheral blood cell; ANC, absolute neutrophil count; ALC, absolute lymphocyte count; PLT, platelet; NLR, neutrophil-to-lymphocyte ratio; PLR, platelet-to-lymphocyte ratio.

Supplementary Table S2. PFS analysis according to CTC and PBC counts.

	Median PFS (95% CI)	*p*-value	HR (95% CI)	*p*-value
CTC C0		0.441		0.443
No ($n = 13$)	12.4 (3.2–21.7)		1 (reference)	
Yes ($n = 34$)	13.5 (6.8–20.2)		0.73 (0.33–1.62)	
CTC cluster C0		0.441		0.443
No ($n = 25$)	15.0 (11.8–18.2)		1 (reference)	
Yes ($n = 18$)	17.1 (NR)		0.73 (0.32–1.64)	
CTC C1		0.387		0.393
No ($n = 7$)	NR (NR)		1 (reference)	
Yes ($n = 33$)	13.1 (8.5–17.6)		1.69 (0.51–5.63)	
CTC cluster C1		0.095		0.101
No ($n = 24$)	15.1 (6.1–24.2)		1 (reference)	
Yes ($n = 16$)	11.0 (8.7–13.3)		1.89 (0.88–4.04)	
CTC C0 to C1		0.707		0.708
Cleared ($n = 6$)	10.0 (NR)		1 (reference)	
Residual ($n = 31$)	13.5 (9.0–18.1)		1.26 (0.38–4.23)	
CTC cluster C0 to C1		0.032		0.037
Cleared ($n = 23$)	27.8 (9.8–45.7)		1 (reference)	
Residual ($n = 14$)	11.0 (8.8–13.1)		2.37 (1.05–5.34)	
CTC cluster C0 to C1 [DC]		0.034		0.047
Cleared ($n = 11$)	NR (NR)		1 (reference)	
Residual ($n = 8$)	9.5 (6.3–12.8)		3.54 (1.02–12.28)	
CTC cluster C0 to C1 [CCRT alone]		0.359		0.364
Cleared ($n = 12$)	8.5 (0.0–22.8)		1 (reference)	
Residual ($n = 6$)	13.5 (8.1–18.9)		1.67 (0.55–5.04)	
ANC C0		0.253		0.256
Low ($n = 25$)	15.1 (10.7–19.5)		1 (reference)	
High ($n = 25$)	11.7 (8.6–17.5)		1.50 (0.75–3.02)	
ALC C0		0.377		0.379
Low ($n = 25$)	8.7 (6.5–11.0)		1 (reference)	
High ($n = 25$)	15.1 (11.0–19.3)		0.73 (0.37–1.47)	
PLT C0		0.609		0.610
Low ($n = 26$)	13.1 (7.6–18.5)		1 (reference)	
High ($n = 24$)	12.4 (7.0–17.8)		1.20 (0.60–2.40)	
NLR C0		0.213		0.217
Low ($n = 25$)	15.1 (10.7–19.5)		1 (reference)	
High ($n = 25$)	9.5 (3.9–15.1)		1.56 (0.77–3.17)	
PLR C0		0.655		0.656
Low ($n = 25$)	15.0 (11.4–18.7)		1 (reference)	
High ($n = 25$)	12.4 (7.0–17.8)		1.17 (0.58–2.38)	
ANC C1		0.681		0.682
Low ($n = 25$)	12.4 (8.8–16.1)		1 (reference)	
High ($n = 24$)	15.0 (12.1–18.0)		1.16 (0.57–2.35)	
ALC C1		0.851		0.851
Low ($n = 25$)	15.1 (7.4–22.9)		1 (reference)	
High ($n = 24$)	12.7 (6.6–18.9)		1.07 (0.53–2.17)	
PLT C1		0.174		0.179
Low ($n = 25$)	17.1 (7.9–26.3)		1 (reference)	
High ($n = 24$)	12.4 (5.1–19.8)		1.63 (0.80–3.31)	
PLT C1		<0.001		<0.001
≤252 x 10^3/μL ($n = 38$)	17.1 (13.4–20.8)		1 (reference)	
>252 x 10^3/μL ($n = 11$)	5.9 (3.2–8.6)		4.15 (1.89–9.11)	
PLT C1 [DC]		0.027		0.039
≤252 x 10^3/μL ($n = 18$)	15.0 (NR)		1 (reference)	
>252 x 10^3/μL ($n = 5$)	5.2 (3.2–7.2)		3.54 (1.07–11.71)	
PLT C1 [CCRT alone]		0.003		0.007
≤252 x 10^3/μL ($n = 20$)	17.1 (13.7–20.5)		1 (reference)	
>252 x 10^3/μL ($n = 6$)	5.9 (0.0–13.0		4.88 (1.55–15.40)	
NLR C1		0.666		0.667
Low ($n = 24$)	13.5 (7.9–19.1)		1 (reference)	
High ($n = 24$)	13.1 (6.0–20.1)		1.17 (0.57–2.40)	

PLR C1		0.277		0.280
Low (n = 24)	15.0 (8.7–21.4)		1 (reference)	
High (n = 24)	9.5 (4.1–15.0)		1.48 (0.73–3.01)	

PFS, progression-free survival; CTC, circulating tumor cell; PBC, peripheral blood cell; CI, confidence interval; HR, hazard ratio; NR, not reached; DC, durvalumab consolidation; CCRT, concurrent chemoradiotherapy; ANC, absolute neutrophil count; ALC, absolute lymphocyte count; PLT, platelet; NLR, neutrophil-to-lymphocyte ratio; PLR, platelet-to-lymphocyte ratio.

Supplementary Table S3. OS analysis according to CTC and PBC counts.

	**Median OS (95% CI)**	***p*-value**	**HR (95% CI)**	***p*-value**
CTC C0		0.910		0.910
No (n = 13)	21.0 (NR)		1 (reference)	
Yes (n = 34)	22.7 (NR)		0.95 (0.40–2.28)	
CTC cluster C0		0.896		0.896
No (n = 25)	NR (NR)		1 (reference)	
Yes (n = 18)	22.7 (NR)		1.06 (0.45–2.52)	
ANC C0		0.002		0.004
Low (n = 25)	NR (NR)		1 (reference)	
High (n = 25)	19.4 (17.5–21.4)		3.46 (1.49–8.00)	
ALC C0		0.053		0.059
Low (n = 25)	19.4 (18.2–20.7)		1 (reference)	
High (n = 25)	NR (NR)		0.47 (0.21–1.03)	
PLT C0		0.558		0.559
Low (n = 26)	NR (NR)		1 (reference)	
High (n = 24)	21.5 (16.5–26.5)		1.26 (0.58–2.72)	
NLR C0		<0.001		0.001
Low (n = 26)	NR (NR)		1 (reference)	
High (n = 24)	19.0 (12.3–25.7)		4.68 (1.95–11.26)	
PLR C0		0.299		0.302
Low (n = 25)	NR (NR)		1 (reference)	
High (n = 25)	21.0 (17.6–24.4)		1.51 (0.69–3.29)	
PLT C1		0.012		0.016
≤252 x 10^3/μL (n = 38)	NR (NR)		1 (reference)	
>252 x 10^3/μL (n = 11)	19.0 (8.9–29.1)		2.72 (1.21–6.14)	

OS, overall survival; CTC, circulating tumor cell; PBC, peripheral blood cell; CI, confidence interval; HR, hazard ratio; NR, not reached; DC, durvalumab consolidation; CCRT, concurrent chemoradiotherapy; ANC, absolute neutrophil count; ALC, absolute lymphocyte count; PLT, platelet; NLR, neutrophil-to-lymphocyte ratio; PLR, platelet-to-lymphocyte ratio.
